# Letter to the editor concerning: Catastrophic apical ballooning in obstructive hypertrophic cardiomyopathy patient treated with mavacamten

**DOI:** 10.1093/ehjcr/ytaf564

**Published:** 2025-11-01

**Authors:** Mark V Sherrid, Bette Kim, Daniele Massera

**Affiliations:** Hypertrophic Cardiomyopathy Program, Leon Charney Division of Cardiology, NYU School of Medicine, NYU Health, 530 First Avenue, Suite 9N, New York City, NY 10016, USA; Hypertrophic Cardiomyopathy Program, Leon Charney Division of Cardiology, NYU School of Medicine, NYU Health, 530 First Avenue, Suite 9N, New York City, NY 10016, USA; Hypertrophic Cardiomyopathy Program, Leon Charney Division of Cardiology, NYU School of Medicine, NYU Health, 530 First Avenue, Suite 9N, New York City, NY 10016, USA

De Roeck et al. present a case of chronic obstructive hypertrophic cardiomyopathy (OHCM), catastrophic acute apical ballooning, caused by severe left ventricular outflow obstruction (LVOTO), cardiogenic shock, and death.^1^ This patient had been treated with mavacamten, and its possible role in the patient’s demise is discussed. Acute apical ballooning mimicking acute myocardial infarction is now understood to be part of the natural history of OHCM, albeit uncommonly, occurring in 1% of cases.^2,3^ Ballooning occurs when latent obstruction becomes severe and unrelenting, and contractility fails, due to afterload-mismatch and supply–demand ischaemia. It does not occur in non-OHCM because these pathophysiologic features are absent. As part of the natural history, acute ballooning had been previously reported in two OHCM patients treated in the Explorer study of mavacamten,^4^ with De Roeck et al.’s case marking the third report.

Discussion in present case report highlights devilish difficulty of decision-making when cardiogenic shock occurs. We have reported on 14 cases of ballooning and cardiogenic shock in OHCM patients due to sudden development of severe LVOTO.^5^ First efforts should be to correct underlying precipitant, be it dehydration, emotion, or acute medical illness. Second, we have administered IV metoprolol 15 mg slowly every 4 h; blood pressure support is provided by phenylephrine, never by positive inotropes. If shock proves resistant, then mechanical support is indicated with ECMO as preferred modality. It is not reported in present case whether LVOTO recurred upon weaning from ECMO and patient’s subsequent collapse, but we have observed this in our case series. When urgent surgery (five patients in our series) was performed to relieve acute LVOTO, we have observed rapid resolution of LV dysfunction within hours and reversal of shock and prolonged survival. This is the most persuasive evidence (among others^2,3,6^) that LVOTO is the cause of ballooning and not an epiphenomenon. There is natural reticence to operate on critically ill patients with LVOTO and shock, but such intervention has proved life-saving; hence, importance of familiarity with this syndrome and a prepared team.

Recognition that a proportion of patients with acute LV ballooning have severe LVOTO and OHCM is an important diagnostic distinction from ballooning patients without obstruction, which we have referred to as neurohumoral Takotsubo syndrome^2,6^ (*Figure 1*). There is considerable phenotypic overlap between the two causes of ballooning, both occurring in elderly females, often with provocations of emotion or medical illness, similar echocardiographic appearance, and both precipitated by catecholamine excess. In former, it is acute LVOTO, while in latter, it appears to be direct catecholamine toxicity to cardiomyocytes or microvascular ischaemia.

**Figure 1:**
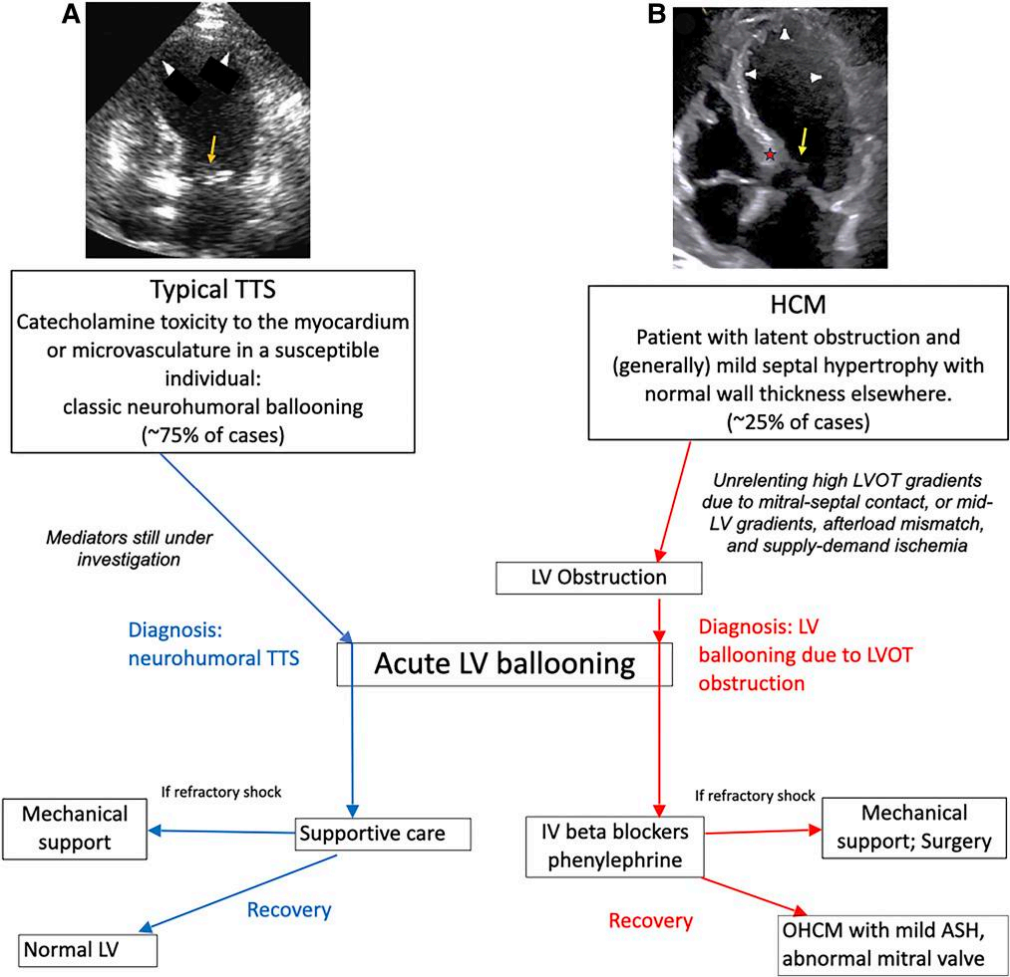
Patients with left ventricular ballooning may have obstructive hypertrophic cardiomyopathy and the sudden development of severe latent obstruction as the cause. Though left ventricular ballooning from obstructive hypertrophic cardiomyopathy and neurohumoral Takotsubo syndrome share similarities, their cause and treatment differ. The presence of septal thickening, systolic anterior motion, high left ventricular outflow tract gradients, and distinctive mitral valve abnormalities in the former distinguish the two. In the recovery phase, the former patients still have obstructive hypertrophic cardiomyopathy while the latter patients have normal left ventricles and normal mitral valves.^2,6^ *Panel* (*A*) Systolic four-chamber echocardiogram in a patient with neurohumoral Takotsubo syndrome. The arrowheads show the ballooned, akinetic apical walls. The orange arrow shows that the mitral valve coapts in the annulus and does not protrude into the left ventricle, and there is no systolic anterior motion. In contrast, *panel* (*B*) shows a patient with left ventricular ballooning due to obstructive hypertrophic cardiomyopathy. The red star shows a basal septal bulge. The yellow arrow shows elongated mitral leaflets that protrude into the left ventricular cavity and coapt markedly apical of the mitral annulus.^2^ There is systolic anterior motion and mitral-septal contact. The left ventricular outflow tract systolic gradient is 80 mmHg. ASH, asymmetric septal hypertrophy; IV, intravenous; LV, left ventricular; LVOT, left ventricular outflow tract; TTS, Takotsubo syndrome.

In a study of 44 unselected consecutive patients with acute LV ballooning and normal coronary angiograms seen in our emergency department, a quarter had OHCM as the cause, with relatively subtle septal thickening of 16 mm overshadowed by the dramatic LV ballooning syndrome. Importantly, these patients had septal thickening before their acute event and OHCM chronically after resolution of LV dysfunction. Physicians should be cognizant that acute LV ballooning can have more than one cause and that treatment may differ accordingly.^6^ A proportion of patients with an acute LV ballooning syndrome have HCM with latent obstruction as the cause. These patients should have targeted treatment to relieve obstruction. With such treatment, even if shock supervenes, their syndrome of cardiogenic shock is potentially reversible.

## Data Availability

Not pertinent for a letter to the editor. However, data from our cited studies will be made available upon request to the senior author.
